# An institutional ethnography inquiry of health care work in special education: a research protocol

**DOI:** 10.5334/ijic.1052

**Published:** 2013-09-18

**Authors:** Stella Ng, Rosamund Stooke, Sandra Regan, Kathryn Hibbert, Catherine Schryer, Shanon Phelan, Lorelei Lingard

**Affiliations:** Faculty of Medicine, University of Toronto, Toronto ON, Canada; Research, Centre for Faculty Development, St. Michael's Hospital; Centre for Ambulatory Care Education, Women's College Hospital; Dept. of Speech-Language Pathology; Faculty of Education, Western University, Althouse College, London ON, Canada; Arthur Labatt Family School of Nursing, Western University, London ON, Canada; Faculty of Education, Western University, Althouse College, London ON, Canada; Professional Communication, Ryerson University, Toronto ON, Canada; Faculty of Rehabilitation Medicine, Department of Occupational Therapy, University of Alberta, Edmonton AB, Canada; Department of Medicine; Centre for Education Research & Innovation, Schulich School of Medicine & Dentistry, Western University, London ON, Canada

**Keywords:** institutional ethnography, special education, children, disabilities, health professional education, joint working

## Abstract

**Background:**

Special education for children with chronic health conditions or disabilities requires the integration of health care work with education. This phenomenon occurs in an understudied and challenging context for integrated care despite policies and protocols that outline work processes in this context. We are interested in an approach to inquiry that will allow us to address gaps in current literature and practices in integrated care, and move towards informing policy.

**Study design and data collection methods:**

Institutional ethnography is an approach to inquiry that maps the actualities of what individuals do at an everyday local level, while examining this work activity in relation to the sociopolitical context. It has been used to change policy and local practice by highlighting disjunctures between policy and actuality. We are adopting institutional ethnography and its three common methods of data collection: document collection, interviews, and observation/shadowing. Informants to this inquiry are chosen from school-based teams, family-centred units and constellations of clinical professionals.

**Methods of analysis:**

We are following work processes, verbally and visually mapping what is done and by whom. It is important to note that work includes ‘unofficial’ work, including the work of families and others who may not be assigned an official work role in a policy or protocol. The mediating role of texts in work processes is also being mapped in order to link the local work to the high-level social coordinators. To begin, analysis focuses on local, or micro-level, work processes; next, analysis identifies and explains the macro-level coordination of the local work (i.e. social and political structures).

**Conclusion:**

A primary outcome of this study will be the creation of verbal and visual maps that demonstrate the social organisation of work processes occurring in the health care-special education interface. These maps will make invisible work visible, highlight disjunctures between policy and practice and identify opportunities for change. They will be useful for critical knowledge translation purposes, providing parents and professionals with an awareness of how their individual work fits in to the larger picture of integrating health care work in special education.

## Introduction

Health-related, school-based support for children with disabilities or special needs requires the integrated working of families, health care professionals and educators. Integrated working is defined as coherent and coordinated service delivery to individual service users across a broad range of health and social care organisations, professionals and informal caregivers [1]. Yet integrated working between health care and education continues to be challenging. In this article, we introduce institutional ethnography, a theoretically informed and practitioner-friendly approach to understanding work organisation [2–7]. Using the example of research we are currently conducting, we propose that institutional ethnography can support the development of knowledge for revising integrated work processes between health care and other sectors such as education.

Our guiding research questions are the following: When health care knowledge needs to be brought to bear on special education, what work processes are occurring and who is doing this work? What documents coordinate/mediate integrated working in special education and how do they coordinate/mediate the work of the team? How is integrated working influenced by social, political and structural relations? Our purpose is to support the improvement of professionals’ integrated working at the nexus of health care and special education, towards a reduction in the burden and inequity experienced by families with children who have disabilities or special needs.

We have chosen institutional ethnography to address these research questions and achieve this purpose. Institutional ethnography uses similar data collection methods as other social scientific approaches - interviews, observations and document analysis - yet it differs in its purpose and focus for analysis. Institutional ethnography is premised on praxis, accomplishing social change for disempowered individuals, by revealing the large-scale coordinators of local, everyday work [4].

We will now introduce some key terms in the institutional ethnography approach to inquiry. First, ‘ruling relations’ or ‘relations of ruling’ refers to how social relations carry out work associated with governance, often without seeming to govern [2–4,8–11]. The term ‘institution’ denotes a metaphorical bundle of social relations that cluster around and coordinate specific societal functions such as health care and education [2–4,8–11]. This definition of institution allows the institutional ethnographer to examine work associated with more than one institution in the context of one local site or even in the context of one work process. Special education, for example, is organised by relations that arise in the economy as well as in education and health care. This conceptualisation of ‘institution’ allows the researcher to grasp that the ruling relations are constituted by, in some cases, networks of agencies or organisations (e.g. for school health or special education, the institution consists of clinics, community centres, schools and school boards, rehabilitation centres, etc.) which together make up an institution.

The work process discussed in this article is the consideration, creation and enactment of a key text: the Individual Education Plan. Specifically, we will focus on Individual Education Plans under construction for children with health conditions or disabilities. The Individual Education Plan is a textually and discursively mediated routine work process employed in special education in much of the developed world. Its stated purpose is to meet the special educational needs of a child for whom the regular educational programme is inaccessible or inappropriate [12–15]. The Individual Education Plan process calls on health care professionals, parents and educators to work together to make recommendations for accommodations, modifications, placements, supports and services to meet the child's needs. Note that ‘work’ in institutional ethnography includes informal and unpaid work. Parents, teachers and health care professionals have all expressed concerns that despite all the work that goes into the Individual Education Plan process, their knowledge is not recognised or utilised effectively and efficiently [12,15,16]. Consequently, Individual Education Plan meetings and their associated outcomes can be tense and unproductive [12,15,17].

Clearly, there is a need for policies and protocols to optimise the success of integrated team work. However, where policies and protocols exist, they risk glossing over the nuances and details of what actually happens in context. Indeed, many integrated care problems are tacit, hidden from view to onlookers and poorly understood by practitioners who may sense that something is amiss but struggle to articulate the nature of their concerns. Understanding the nature of this phenomenon is crucial to the development of children and youth with disabilities. Awareness of the actualities of this work process is also crucial to the health and education systems; professionals expend valuable resources by engaging in ineffective practices at the health care-education junction [17–19].

## Integrated systems and team working: brief summary of literature

The United Kingdom (UK) has been a leader in research on integrated working. Integrated working (commonly referred to in the UK as joint working) has been both a policy focus and a practical problem in the UK since the 1970s [1]. In Canada, interprofessional collaboration and family-centred care [20,21] have arisen along the same time frame. However, because IPC and FCC do not explicitly emphasise inter-sector and inter-system complexities, we do not situate our research within these bodies of literature.

The existing literature on integrated working has focused more heavily on the health care-social services junction rather than on the health care-education junction [22–24]. Although policies have been in place to promote integrated working for several decades, problems persist and needs continue to increase [1]. The majority of research on integrated working focuses on general perceptions of what works and what does not work, as reported by professionals and in fewer cases, parents [19,25]. Our review of the extant literature suggests the following perceived barriers to integrated working: ill-defined or discrepant understandings of roles and responsibilities across organisations and professions, differences in attitudes and values across organisations or professions, and ineffective communication practices [1,19,22,26–32].

Culture - rather than structure - has been identified as one of the most powerful perceived influences on the success or failure of integrated working efforts [24,29]. We are using culture as a broad term to describe a number of factors identified in the literature review, which will be discussed below. These factors include the following: organisational and professional culture, professional identity and documentary practices. Documentary practices are the ways in which documents constitute work. In special education, health care professionals’ documentary practices are in some cases the primary or even sole means of communication and representation from a particular discipline [17,33]. How these documents are taken up in the educational culture is a complex problem related to institutional and professional culture. For example, in a study of collaborative relationships between psychologists and speech-language pathologists who shared goals for children with special language learning needs, documentary communication practices were implicated as the main barrier to successful collaboration [33].

### Documentary practices in integrated working

The written practices of health care professionals across organisations are reflective of higher systemic policies and regulations and of organisational and professional culture [4,34]. Documents play a role in the representation and development of organisational knowledge; they are active constituents of social relations [4,35]. Consider the path of documentation from health care to education. Health-related recommendations for the educational setting are informed by health care professionals’ assessment results and recommendations in the form of a written report [36]. Health care professionals’ reports are governed by diagnostic manuals, professional report-writing conventions, college/regulatory documents and policy documents. The health care professional report, once transferred to the educational setting, acts as or mediates the health care professional's voice and exerts influence on the education professionals’ subsequent actions.

Documentation for children to receive special education services is mandated in education policy but requires assessments from health care professionals [36,37]. Thus education policy and discourses as well as health care policy and discourses mediate the practices of health care professionals at the education–health care interface. While health care professionals’ recommendations are meant to be translated into educational programming, health professions’ discourses (e.g. the medical conceptualisation of disability) and assessment standards may or may not align with education discourses (e.g. educational exceptionality) and assessment standards [17]. Further, the need for and power of documentation plays a role in families’ everyday work to support their children, and potentially in children's identities [38]. For example, if in order to access support a child must have a documented condition, this requirement may shape the professionals,’ family's, and child's perception of the child's disability and identity. This complex, multidirectional, textually and discursively mediated process requires a robust investigation.

### The gaps in the literature

The review of literature suggests that although policy and structural support is in place to support integrated working, there are persistent barriers to the realisation of integrated working, particularly at the health-education juncture for children. Thus a practical, mutual understanding and critically reflective [38] process is required across all levels of stakeholders [29]. Effective integration may rely on cultural factors even more than on structural or systemic changes [24,29]. As mentioned above, culture is related to professional identity and socialisation and is also enacted and shaped by documentary practices [39]. Further, the focus of most relevant literature is on the health care-social services junction. Little is written about the junction between health care or social services and education services, despite the fact that school-age children spend a large proportion of their waking hours in school.

### Context of our study: families’ experience of special education in Canada

A Statistics Canada report from 2008 demonstrated an increased prevalence of disability in children of 4% between 2001 and 2006. This same report stated that nearly one-quarter (24.3%) of parents of children with disabilities reported that their children were not receiving necessary special education services. Of this group, nearly half of the children had severe or very severe disabilities and nearly two-thirds had undergone professional assessment of their educational needs. Nearly half of children requiring special education (49%) had parents who reported difficulty in accessing special education services while two-thirds (64%) of parents of children with very severe disabilities reported challenges obtaining special education for their child [40–42]. The majority of children with disabilities does not attend special education classes but, rather, is integrated into regular classrooms [40–42].

This ‘inclusive’ model of special education further demands the work of families and education and health care professionals to ensure that children with disabilities can function in the regular classroom setting through accommodations and modifications. Reasons reported by parents that outline insufficient services for children with disabilities included the following: insufficient staffing or services, difficulty obtaining necessary assessments from health care professionals, communication problems and a lack of access to resources locally [40–42]. These reasons as well as the many challenges of integrated working, as we have outlined above, may limit the extent to which systems are truly ‘inclusive.’ Provincial and national agencies have identified a need for practice- and research-based investigations into strategies to improve integrated working [43,44].

## Institutional ethnography: why and how?

Institutional ethnography is well-suited to address the challenges of integrated working. It shares with integrated care an ethical imperative to support people whose circumstances oblige them to navigate complex institutional landscapes. Moreover, like professionals providing integrated care, institutional ethnographers view the coordination of work as a work process in its own right. Institutional ethnography is, in fact, a strategy for examining coordination as work. Thus it will allow us to examine the intentional coordination that goes on in integrated care settings and the unintentional coordination that happens as people from all walks of life go about routine activities in workplaces and elsewhere. The primary goal of institutional ethnography is to show how the work involved in a specific process, such as the Individual Education Plan, is being coordinated via texts and by discourses [9].

Institutional ethnography was created by the Canadian sociologist, Dorothy Smith [4]. Smith adopted an ontological assumption from ethnomethodology to propose that ‘the social’ is a web of coordinated activity. She adapted Marx's notion of materialism and the social relation to propose that the coordination of social activities is accomplished in sequences of actions that link the action of one individual with the actions of others. In the creation of an Individual Education Plan, for example, social relations link the work of health care professionals to the work of parents and classroom teachers.

Consider what happens when a health care professional introduces a commercially produced, norm-referenced assessment protocol to the Individual Education Plan process. Contributing the assessment data ‘hooks’ the health care professional's work (and by extension the work of the team) into a web of relations that transcends the local context of the health care professional's practice. Institutional ethnographers working in the field of educational policy have mapped ways in which commercially produced assessments and support materials for teachers and families are organising and standardising educational work in schools and homes across continents [45]. Salient to the current discussion is that the standardisation of practices across diverse settings took place in the absence of regulation [45]. The findings show how participating in the extra-local relations and organising work in a local site subordinates the participant's goals to those of others elsewhere.

### Work in institutional ethnography

As mentioned in our introduction, we are focusing on the work that goes into considering, creating and implementing an Individual Education Plan. Institutional ethnography's generous definition of work allows researchers to view managing an illness, advocating for a child at a team meeting, expressing anger, even feeling anxious, as work and to consider how such work contributes to the situation being investigated. For example, writing about changes in long-term care in the wake of a total quality management exercise, Campbell [46] identified ‘a creeping colonization of minds and hearts of the caregivers with the goals and values of the market’ (p. 93). Some Individual Education Plan activities do not fit easily into standard work categories. Without the work of families, for example, an Individual Education Plan process cannot proceed. Equally important, however, are texts and discourses. In post-industrial societies, work processes are increasingly mediated by texts and discourses [2,4,11,47,48].

### Texts and discourses in institutional ethnography

When people engage with particular texts and participate in certain discourses, their work may be entered into social relations that articulate their local actions to the ruling relations. Institutional ethnography pays particular attention to the mediational affordances of replicable texts both print and digital formats because ‘anyone else anywhere else can read, see, hear, and so on, the same words, images, or sounds as any other person engaged with the same text’ [p. 66, 4]. Protocols for integrated working and curriculum documents, for example, have capacity to organise work in local settings such as public health agencies and schools. Indeed, their purpose is often to standardise routines across organisations [4,35].

Certain collaboratively developed texts such as patient case files and Individual Education Plans mediate action in a more complex fashion. They are created over time, often in steps or stages, and are used for a variety of purposes and in sequences of action, in more than one work setting [4,35]. By investigating such textually mediated work processes in fine detail, institutional ethnography inquiries can bring into view places where the original purpose of a work process is being undermined or subverted to another (implicit) purpose.

### Finding a problematic and adopting a standpoint: the entry point for an institutional ethnography

To understand the impact of ruling relations, institutional ethnography investigations adopt an explicit standpoint; in our case, we have adopted the standpoint of families. The problematic - the point of disjuncture between actualities of experience and intentions of protocols and policies - is the inefficiency and inequity in the process of considering, creating, implementing and refining/revising an Individual Education Plan for a child. We will now explain these key concepts of institutional ethnography in more detail, beginning with the problematic and moving on to the standpoint.

Local forms of work organisation are generally visible to the people who do the work; however, extra-local relations are rarely entirely visible to people going about routine activities. For example, teachers and health care professionals may be aware that Individual Education Plan recommendations are coordinated with school board assessment practices, yet unaware of the extent to which ideas about ‘normal achievement’ are mediated by Canada's participation in international assessment programmes such as the Organisation for Economic Cooperation and Development's Programme for International Student Achievement. Institutional ethnographers have shown how ‘conceptual currencies at play in any historical moment are picked up across institutional complexes and woven together in mutually reinforcing ways’ [p. 296, 8], but for people going about routine activities these forms of coordination can create situations often as mystifying as they are frustrating.

In institutional ethnography, people's experiences of frustration and mystification point to the existence of a problematic [4]; that is, a question or questions that are latent in a situation but have not yet been articulated by the people whose experiences are shaped by the problematic. Institutional ethnographers thus begin inquiries by exploring the local contexts in which a problematic is being experienced with a view to identifying a work process implicated in the production or maintenance of the situation. The work process then becomes the focus of detailed exploration in order to identify points at which individuals’ actions are being hooked into the ‘ruling relations.’ Our entry point is the Individual Education Plan process - from early consideration of potential need for an Individual Education Plan to the refinement/monitoring stages of an Individual Education Plan enacted.

The notion of standpoint has been the topic of ongoing debate in qualitative research literature [4]. Some critics argue that ‘the standpoint of women’ essentialises women's experiences, appropriates the experiences of some women to serve the interests of others, and uncritically accepts accounts of personal experiences as true [p. 91–92, 46]. In response to these criticisms, institutional ethnographers assert that a standpoint in institutional ethnography does not imply the adoption of a perspective but a position with respect to the ruling relations. Taking a standpoint with people who participate in the ruling relations, but not as agents, affords a view of the ruling relations that is not so much bottom-up as outside-in [49]. They note too that people's accounts are not treated as windows on participants’ experiences. Rather, they are sources of clues about work organisation and starting points for tracing linkages between work carried out in one setting and work carried out by another [50].

It is important to note that institutional ethnography's mode of inquiry sets it apart from other social scientific approaches such as grounded theory, phenomenology and other forms of ethnography. Institutional ethnography inquiries employ data collection methods routinely employed in sociological field work [49], but the analysis, which begins early in an inquiry, more closely resembles modelling, a strategy employed extensively in natural science research. Institutional ethnography does not rely solely on inductive or deductive analysis. It makes no claim to objectivity and does no attempt to generalise findings but proceeds by following up on clues about the coordination of a specific work process and producing maps of institutional terrain for people to use [11]. Hence, while people's experiences do provide the entry points for inquiries and participants often share their subjective perspectives in interviews, institutional ethnography inquiries attend to what people are actually doing in relation to a situation, not to their subjective views [2].

### The Individual Education Plan process as entry point and the family as standpoint: justification

A student's Individual Education Plan, like a patient's case file, seeks to coordinate the actions of diversely situated practitioners and families. The Individual Education Plan process is initiated when a child's educational needs have been shown to be out of sync with the curriculum expectations for that child's age-determined grade level. The Individual Education Plan document identifies the learning expectations for the student, outlines how the school will address the expectations by implementing appropriate special education programmes and services and notes how the student's progress will be reviewed [36,37,51]. The Individual Education Plan is developed and modified on the basis of continuous evaluation and assessment by education and health care professionals from within and outside the school system. Resource documents assist school board officials, principals, teachers, students and their families, health care workers and community workers in meeting the planning and regulatory requirements for students with an Individual Education Plan [36]. Research on the Individual Education Plan has demonstrated that it can be confusing, frustrating or ineffective if non-specific or unproductive language (e.g. sweeping statements and unsubstantiated rhetoric) is used, if parents are unsatisfied, and if teachers do not ‘buy in to the plan [12–15]. As noted in the introduction, the Individual Education Plan and its associated processes can be troubling for all concerned. For this reason, it is a good entry point for an institutional ethnography investigation into the social organisation of the special education–health care interface.

Related to the Individual Education Plan, a School Support Team (SST) or other similarly ‘purposed team includes parents, education professionals and health care professionals from within and external to the school. At School Support Team meetings, School Support Team members including parents meet to discuss the multiple assessment findings and recommendations of health care and education professionals. These discussions guide the development and the modification of the Individual Education Plan. We are chiefly interested in Individual Education Plan processes, including early discussions about whether an Individual Education Plan is appropriate or needed, that draw substantively from health care professional input.

We recognise that families and professionals of all types contribute to the creation and implementation of an Individual Education Plan. There is a need to better understand how their work in school-based integrated care is being organised locally and extra locally. We have adopted the standpoint of the child's family because our pilot data confirm that it is the family who must travel across systems, often translating and delivering messages from professional to professional, across organisational/professional boundaries. For these reasons, taking the standpoint of the family can provide critical information to inform and improve health care professional practice at the health care-education junction. And ultimately, the standpoint of the family allows us to use this inquiry to strive towards equitable care, services and supports *for* families. It is important to note that despite our family-oriented standpoint, multiple informants will contribute to the explication of the work processes. These other informants will include education and health care professionals, administrators, families and policy-makers

## Methods

### Study location

Four publicly funded school boards in Ontario, Canada, will serve as our education settings. By recruiting across boards, not only will we be better able to distinguish board-specific nuances from policy-imposed structures, but we will also reduce the burden imposed on each board thus facilitating participant recruitment.

### Informant selection (sampling) strategy

Two main methods resulting in three ‘units’ of informant selection will be pursued. First, eight schools (two from each of the four school boards) will be recruited for participation. Within each school, school-based data collection focuses on school-based sampling units. In addition to school-based informant selection and data collection, at each school, one consenting family will be shadowed closely over time as they navigate the complex systems of professionals and processes, forming family-centred shadowing units. Finally, in order to capture the broader constellation of sectors, agencies and professionals, ‘constellation data’ will be collected. Essentially, constellations of clinically based professionals will be interviewed based on purposive sampling guided by analysis of school-based data. A more detailed description of each type of informant selection follows.

First, we explain our school-based informant selection units. Each school will serve as a source of data. Each school has a team of static members, members who are always a part of the overall school team and also dynamic members, members who change based on the family that the team strives to support.

Next, we explain our family-centred shadowing units. From each of the eight schools that we will use as an informant selection unit, we will recruit and closely follow one family (for a total of eight families) as they navigate through the health care-education interface. We will collect documents that the families share with us on an ongoing basis, interview family members and consenting professionals involved over the one- to two-year period, and observe team meetings over the one- to two-year period.

Finally, we explain our selection of informants from the constellation of individual clinical professionals in the local communities of the school boards. To supplement the detailed and context-specific data we will obtain from following these eight families, we will also recruit from the broader constellation of professionals who navigate the health care-education interface, with informant selection based upon the analysis of the family-centred data (purposive sampling). These data will take the same form: additional documents, interviews of additional professionals and observation of additional team meetings. These data will serve to expand the view of the health care–education interface and gain multi-perspective insight into how health care and education interact to support children with disabilities.

### Data collection procedures

Participating school boards have connected us with gatekeepers to guide the selection of schools from which to sample. Data collection will occur over the period of two school years, a time frame we think is appropriate based on how our pilot data collection is progressing. Details of our three data collection methods - observation/shadowing, document collection, interviews - are provided next.

First, we will conduct observation of an initial School Support Team meeting and other meetings and interactions and shadowing of other appointments/meetings. Observations will be of School Support Team meetings (one initial meeting at which the student's needs are first formally discussed and one follow-up meeting to revisit plans and discuss progress, per School Support Team) and shadowing of other appointments or meetings on a purposive and consented basis. Shadowing is an observational method, which involves a researcher closely following a subject over a period of time to investigate everyday work [10]. The importance of observation and shadowing is uncovering the realities of everyday practices; by only analysing documents or interviewing participants, there is a risk of merely rearticulating institutional ideology [10]. Additional related meetings will also be observed on a purposive basis. The observational and shadowing data will include detailed ethnographic fieldnotes [52] not only on work processes but also on the contexts in which integrated working is occurring. Observations will only occur with consent, as per our research ethics board-approved process, from all individuals present.

Second, we will collect key documents. Documents that will be collected include the following: policy (e.g. the Ministry of Education IEP Standard, Human Rights standards for accessibility for individuals with disabilities), community initiatives (local networks, public health and community protocols, and school board's locally developed protocols), school documents (Individual Education Plan development meetings and minutes), and local clinical/hospital professional documents (professionals’ reports). In institutional ethnography, a ‘chain’ of texts and work processes may be identified by attending to the connections that informants reveal in their interviews or work [9]; therefore, we expect to sample additional text types, following our participants’ lead.

Document contributions will be invited from all participants. Parents are expected to be the main contributors of professional reports about their children, for privacy reasons and as discussed with our school board sites. Documents not provided directly by parents will not relate to any of the families included in our study and will be completely de-identified. For example, a teacher may provide a thoroughly de-identified Individual Education Plan, but not one that is for a student that we may observe while at the school. Parents will be fully informed of their choices as per our research ethics board-approved informed consent process. This process covers confidentiality and privacy, data security and personal health information protection measures.

Third, we will conduct interviews. Interviewees will be purposively chosen based on the ongoing data collection and analysis process, consistent with constant comparative method that is used in qualitative research methodologies and can be appropriately applied to institutional ethnography [53–55]. In institutional ethnography, interviews serve the primary purpose not of understanding individual experience, as in other qualitative methodologies, but rather of investigating organisational and institutional processes [9]. Questions focus on the practices or work [9] and analyses of interviews focus on identifying trans-local relations, discourses, and institutional processes that are shaping the informants’ everyday work [9]. Informants themselves may not be aware of the forces shaping their everyday work, and it is the researcher's job to discover these forces in the constant interchange between data collection and analysis [4,9]. Professionals will participate in one to two interviews each, and parents in one to three as well as informal interviews as initiated by parents as new developments arise.

### Data analysis plan

NVivo software [56] will be used for organisation and facilitation of analyses. Analysis of the observation and interview data will proceed in an iterative fashion. First, interview transcripts and fieldnotes will be coded (at first, independently by two researchers) for any emerging work processes. Issues for further questioning or further observation will be flagged for follow-up which will be conducted purposively. These data will be viewed in light of the documentary analysis that will be ongoing.

Analysis in institutional ethnography occurs at two main levels. First, work processes are traced at the local level with a detailed explication of the work process. Second, the work processes are related back to a higher level - ruling relations - identifying the social, systemic and political coordinators of local action. By identifying and explicating the coordinators of recursively occurring work, we can understand social organisation - what people are doing and why [4]. For example, in our preliminary data, we found that health care to special education communication occurs mostly through written communication, although experienced health professionals reminisce about days when they could attend School Support Team meetings at schools or make phone calls more readily. In looking ‘up’ to coordinators of this process, privacy legislation, health professional genres of communication, budget and billing constraints, and time and human resource factors were implicated as drivers of this process. Institutional ethnography begins but does not remain in the local; it must, in the analysis phase, look ‘up’ to social relations to explicate social organisation of local work [4,5,11]. Texts serve as ‘clues’ in this process, linking the micro- and macro-level analyses to reveal social organisation because texts are imbued with discursive influences and have a strong mediating role in people's actions [4].

While most data will be analysed and organised in a written format, we will take a closer look at certain foci of the work processes that we uncover and create visual maps [11]. The purpose of these visual maps will be to expose disjunctures between what people espouse to be a work process and what is actually done. For example, our pilot interview data are showing a clear disjuncture between what clinicians believe happens or should happen with their written recommendations, and what actually transpires as that document moves from clinic to school. Indeed, even how and which parts of the document move from clinic to school are not explicitly outlined anywhere but are becoming apparent through our institutional ethnographic approach. Mapping this work process in depth may enable clinicians to more effectively create their recommendations, with the necessary knowledge of the larger work process of which their own work is a part. Additionally, we will create higher-level - as opposed to fine-detail - maps to illustrate the broad inter-system relations, and micro–macro relations.

We suggest that our research protocol provides a well-triangulated data set and rigorous and systematic data analysis approach. We are drawing from three types of data collection methods and a myriad of ‘types’ of individuals as informants to the work process.

### Ameliorating inherent limitations

Although efforts will be made to obtain diverse and representative informants, because participation is voluntary, the purposive sample may, indeed, be in part a convenience sample. However, because we will also collect documents across levels (macro and micro) and because parent participants can provide the written documents of professionals (with professionals de-identified) we will be able to address some of the issues associated with a convenience sample.

The Hawthorne effect is inherent in any observational data collection technique; informants may change their routines because they are being observed [10]. The multiple data collection approaches and prolonged immersion in the field will help ameliorate this problem by filling gaps that just one data collection method would leave and allowing interpretation of potential Hawthorne effects.

We will not be interviewing children, and we are aware that this is a crucial gap. We made this decision based on feasibility and discussion with our school board collaborators, and we do have plans and expertise in place to actively engage children in future work following from this research.

Disabilities studies raise the consideration that in a medical discourse that frames disability as a physiological deficit, individuals with disabilities may be marginalised [38,57]. Disabilities studies are thus relevant to this research, but we will not focus, *a priori*, on the sociological construct of disability. In this protocol, we have used language consistent with the articles we reviewed in identifying the gap and need for this research. Thus disability is described in this article within the frame of a biomedical and biopsychosocial model [58]. However, we acknowledge that in the analysis stages of this research, the social forces described in disabilities studies are likely to be implicated. Future papers explaining findings from this work will address the discursively shaped nature of special education and disability.

## Conclusion

Special education for children with chronic health conditions or disabilities is a critical example of integrated care. Our literature review and piloting of methodology and methods (leading to this article) have suggested that health care professionals, educators and families are caught in uncharted terrain of social organisation. Families, consequently, traverse this terrain without a map, meeting myriad professionals who also lack orientation to the landscape, yet who must provide written documents without always knowing their destination (or audience). Meanwhile, the goal of the journey - meaningful support for children with disabilities or special needs - cannot be readily achieved even with considerable parental and professional effort.

Institutional ethnography is a useful theoretical and methodological approach to gaining understanding of the institutional coordination of complex work processes. In particular, it offers generative possibilities for changing practice by explicating tacit knowledge and invisible work and how these are textually mediated, as well as ultimately discursively or politically coordinated. Institutional ethnography findings can be used to provide a map to families and professionals who are caught up in the complex work processes of integrated care, enabling them to see the ruling relations that mediate their everyday lives. Through such a raised awareness, individuals will be better equipped to exact change and influence in their everyday work.
